# Diagnostic cutoff values of synovial fluid biomarkers for acute postoperative prosthetic joint infection: a systematic review and meta-analysis

**DOI:** 10.5194/jbji-9-17-2024

**Published:** 2024-01-29

**Authors:** Marta Sabater-Martos, Marc Ferrer, Laura Morata, Alex Soriano, Juan Carlos Martínez-Pastor

**Affiliations:** 1 Orthopedic and Traumatology Department, Clínic Barcelona, Carrer Villarroel 170, 08036 Barcelona, Spain; 2 Department of Infectious Diseases, Clínic Barcelona, Carrer Villarroel 170, 08036 Barcelona, Spain; 3 IDIBAPS, CIBERINF CIBER in infectious Diseases, University of Barcelona, Spain

## Abstract

**Introduction**: The assessment of white blood cell (WBC) count and polymorphonuclear cell (PMN) percentage in synovial fluid can help in the diagnosis of acute postoperative peri-prosthetic joint infection (PJI). Their cutoff values, which would differ from those for chronic PJI, have not yet been determined in acute postoperative PJI. The aim of this study was (1) to analyse studies reporting the optimal cutoff values for WBC count and the PMN percentage in synovial fluid and (2) to determine which is the best diagnostic tool for acute postoperative PJI. **Methods**: We performed a systematic review (SR) of primary studies analysing WBC count and the PMN percentage for diagnosis of acute postoperative PJI. A search was performed in MEDLINE and EMBASE. We studied the risk of bias and quality assessment. We extracted data on cutoff values, sensitivity, specificity, positive and negative predictive value, area under the curve, and accuracy. We calculated the diagnosis odds ratio (DOR), performed the meta-analysis and summarized receiver operating curves (sROCs) for WBC count and the PMN percentage. **Results**: We included six studies. WBC count showed a DOR of 123.61 (95 % CI: 55.38–275.88), an sROC with an area under the curve (AUC) of 0.96 (SE: 0.009) and a Q index of 0.917. The PMN percentage showed a summary DOR of 18.71 (95 % CI: 11.64–30.07), an sROC with an AUC 0.88 (SE: 0.018) and a Q index of 0.812. **Conclusion**: We concluded that WBC count and the PMN percentage are useful tests for the diagnosis of acute PJI; WBC is the more powerful of the two. Studies centred on other synovial fluid biomarkers not yet studied could help in this diagnosis.

## 1 Introduction

Arthroplasty replacement surgery is one of the most performed procedures in the orthopaedic field. Unfortunately, infection remains one of the most serious complications in prosthetic surgery, occurring in 0.5 % to 2 % of patients and increasing to 10 % in revision surgeries (Kurtz et al., 2010; Tande and Patel, 2014; Zimmerli et al., 2004; Bauer et al., 2006). This complication involves high costs, both economically and in terms of health resources (Ariza et al., 2008; Kurtz et al., 2008). Peri-prosthetic joint infection (PJI) studies have increased exponentially over the last 2 decades; different consensuses have been reached, advising on the best treatment approaches (Workgroup and Society, 2011; Goswami et al., 2018; Signore et al., 2019). The assessment of synovial fluid biomarkers such as white blood cells (WBCs) and the percentage of polymorphonuclear (PMN) cells are useful in reaching a PJI diagnosis. However, these studies have been centred on chronic PJI. Recent studies suggest that cutoff values for WBC count and the PMN percentage are too low for acute postoperative PJI diagnosis; thus, values for acute postoperative infection should be much higher (Bedair et al., 2011).

It has also been demonstrated that both synovial fluid WBC count and PMN percentage change over time, in both non-infected and infected joint prosthesis (Christensen et al., 2013). However, there is no clear consensus as to where these cutoff values lie. Therefore, acute postoperative PJI lacks clear synovial diagnostic cutoff values, and diagnosis relies on clinical suspicion or on cultures – which can delay treatment.

At this time, there are several points in relation to acute postoperative PJI upon which consensus has not been reached, such as time after surgery to consider an acute infection and the cutoff parameters for diagnosis in synovial fluid. Therefore, the aim of this study was (1) to analyse studies reporting optimal cutoff values for WBC count and the PMN percentage in synovial fluid and (2) to determine which of the two tests is the best diagnostic tool for acute postoperative PJI.

## 2 Material and methods

This systematic review (SR) follows the Preferred Reporting Items for Systematic and Meta-Analysis of diagnostic test accuracy studies (PRISMA-DTA) (McInnes et al., 2018). The protocol for this SR was published on the Prospero database (CRD42021292751).

### 2.1 Search strategy

A computer-aided search was performed on the MEDLINE and EMBASE databases from inception to 29 December 2021, to identify primary studies analysing optimal cutoff values for synovial WBC count and PMN percentage in acute postoperative PJI. The bibliography of each of the included articles was also reviewed to identify additional eligible primary studies. No restrictions were placed on publication dates. Only articles written in English, French or Spanish were considered. The search strategy combined terms related to acute infection (e.g. “acute prosthetic joint infection”, “acute total knee arthroplasty infection”, “postoperative acute infection”), terms related to synovial fluid diagnosis (e.g. “synovial leucocytes”, “synovial white blood cell count”, “synovial neutrophils”) and terms related to diagnosis accuracy (e.g. “sensitivity”, “specificity”). The full electronic search strategy is provided in the Supplement (see Table S1 in the Supplement).

### 2.2 Inclusion and exclusion criteria

Eligibility criteria are described below, according to PICO structure: this SR included diagnostic studies conducted on patients with suspicion of an acute postoperative prosthetic joint infection in which a synovial fluid aspiration with WBC and/or the PMN percentage was performed to determine cutoff values, sensitivities and specificities. We excluded articles that studied diagnostic accuracy in late acute infections or chronic PJI. Studies published on this topic have mainly focused on hip and knee prosthesis and so has this review.

**Figure 1:**
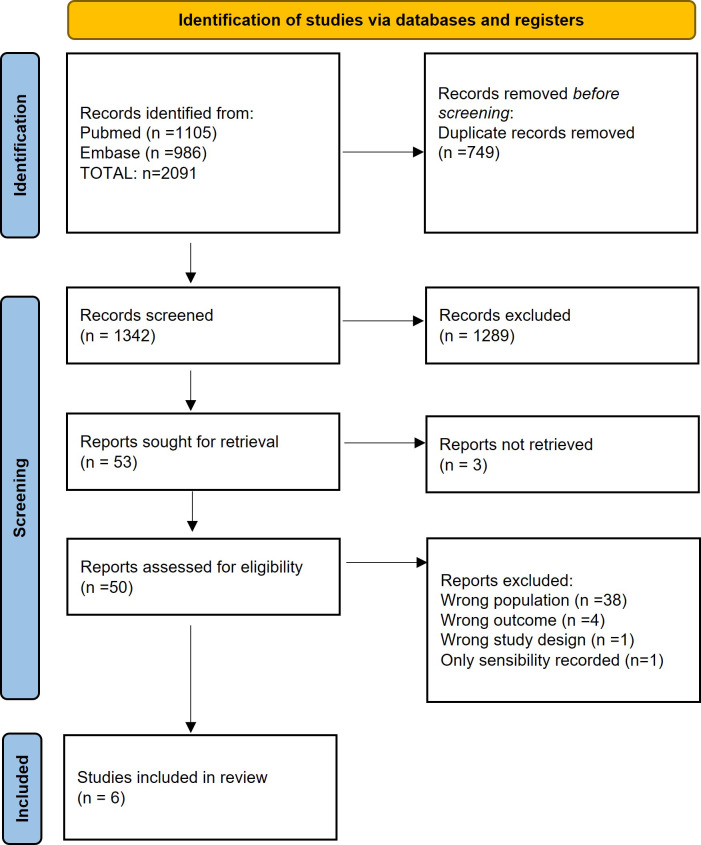
PRISMA 2020 flow diagram for new systematic reviews, which included searches of databases and registers only (Page et al., 2021).

We eliminated duplicates using the Mendeley reference management software. The screening process was done using the Rayyan software (intelligent systematic review). Two independent investigators (Marta Sabater-Martos and Marc Ferrer) did an initial screening by title and abstract and a second screening by complete text reading, following the inclusion and exclusion criteria. Discrepancies were resolved by consensus; when consensus could not be reached, a third investigator provided a decision (Juan Carlos Martínez-Pastor). Figure 1 shows the process of study selection (Page et al., 2021).

### 2.3 Data extraction

Two investigators (Marta Sabater-Martos and Marc Ferrer) extracted data by duplicate. Discrepancies were resolved by consensus. We collected the following data: first author, year of publication, number of patients included, characteristics of the population (age, sex, comorbid conditions), infection diagnostic definition, operated joint, WBC and PMN percentage cutoff values (whether derived from the use of receiver operating curve (ROC) or predetermined), sensitivity and specificity, positive and negative predicted value (PPV, NPV), area under the curve (AUC), and accuracy.

### 2.4 Risk of bias and quality assessment

Two independent investigators (Marta Sabater-Martos and Laura Morata) assessed the risk of bias and the quality of each primary study using the Quality Assessment of Diagnostic Accuracy Studies (QUADAS-2) (Whiting et al., 2011). This tool assesses the quality of studies including analysis of bias risk and study applicability. It consists of four domains: (1) patient selection (methods of patient selection, applicability of included patients), (2) index test (description of conduct and interpretation, applicability of index test), (3) reference standard (description of conduct and interpretation, applicability of reference standard) and (4) flow and timing (exclusion criteria, time interval between index test and reference standard, applicability of patient flow). Each domain was assessed in terms of the risk of bias; the first three domains were also assessed in terms of applicability, with ratings of “low”, “high” or “unclear.” Discrepancies were resolved by a third author (Alex Soriano).

### 2.5 Information synthesis and statistical analysis

The meta-analysis was conducted only on synovial WBC count and PMN percentage. With the information extracted we were able to calculate the diagnostic odds ratio (DOR) for every study. Even though sensitivity and specificity are usually used in diagnostic test studies due to their clinical applicability, when comparing different studies, they can produce what it is called the threshold effect. For this reason, we used the DORs, with 95 % confidence intervals (CIs), and the summarized receiver operating characteristic curve (sROC) for the meta-analysis (de Sousa et al., 2009). A random-effects model and DerSimonian–Laird model were used respectively.

Although the DOR has limited clinical applicability, it becomes useful in diagnostic SR because it is a measurement of the effectiveness of diagnostic testing. It is defined as the ratio of the odds of the test being positive when the patient has the outcome over the odds of the test being positive when the patient does not have the outcome. This measurement ranges from zero to infinity, with values superior to 1 indicating that the test is useful, while larger values indicate better performance in identifying the outcome (Arias and Molina, 2015; de Sousa et al., 2009).

The sROC is an estimation of a common ROC curve for all the included primary studies. We also provided the summarized Q measure and the AUC of our new curve. The Q measure indicates the point in the sROC curve where equal sensibility and specificity are found. This measurement ranges from 0 to 1, with values inferior or equal to 0.5 indicating that the test is not useful; a value closer to 1 indicates better performance in identifying the outcome (de Sousa et al., 2009; Arias and Molina, 2015).

Heterogeneity among the reported sensitivities and specificities was measured with the use of the Higgins $I^{2}$ test. This test describes the percentage of total variation across all the studies that is attributable to heterogeneity rather than chance or sampling error. The Higgins $I^{2}$ value ranges from 0 % to 100 %, where values from 0 % to 40 % indicate that heterogeneity may not be important, 30 % to 60 % may represent moderate heterogeneity, 50 % to 90 % substantial heterogeneity and 75 % to 100 % considerable heterogeneity. All statistical analyses were performed using Jamovi (version 2.2.5) and Meta-disc (version 1-4).

## 3 Results

### 3.1 Search results

The primary search identified a total of 2091 potentially eligible records published up to 29 December 2021. After removing duplicates, we screened 1342 primary studies for title and abstract. Of the remaining 53 studies, we excluded 3 because we were unable to find the study's full text, even after direct contact with the authors in one case; the remaining two were conference or congress abstracts. When the full texts of the remaining 50 studies were screened, we removed 38 for describing different populations, 4 for having different outcomes, 1 for having a different study design and 1 for reporting only sensitivity (see Table S2). After manual review of the bibliographies of the primary studies included, no other studies were identified that met our eligibility criteria. In the end, we included six studies (Bedair et al., 2011; Yi et al., 2014; Kim et al., 2017; Sukhonthamarn et al., 2020; Dugdale et al., 2022; Uvodich et al., 2021) (Fig. 1) (Page et al., 2021).

**Figure 2:**
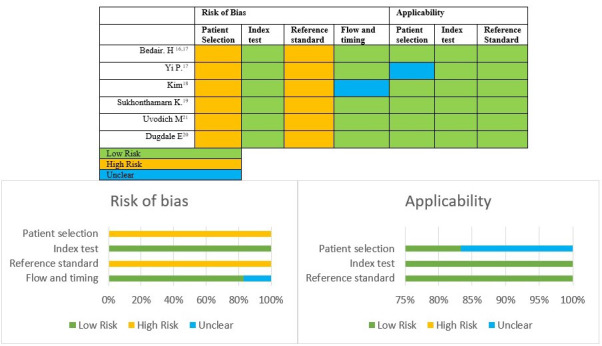
QUADAS-2 tool. Summary of risk of bias and applicability for all included studies.

### 3.2 Risk of bias and quality assessment

We performed the risk of bias and quality assessments using the QUADAS-2 tool (Whiting et al., 2011); results are presented in Fig. 2. Overall, primary studies were classified as having a high risk of bias and good applicability. All studies showed problems in patient selection domains: none provided a clear selection process, and all were retrospective. The reference standard domain also raised important concerns, as the diagnostic reference for acute postoperative PJI is not well defined in the literature, and it has been changed over time (Trampuz and Zimmerli, 2005; Signore et al., 2019; Parvizi et al., 2018; Workgroup and Society, 2011). See Table S3 for more information.

### 3.3 Characteristics of the primary studies

The six primary studies included in this SR were published between 2010 and 2021. They included a total of 826 patients (range 43–197), of which 227 were infected. All studies were case-control diagnostic studies. Three studies assessed only knee joints (Uvodich et al., 2021; Kim et al., 2017; Bedair et al., 2011), two only the hip joint (Yi et al., 2014; Dugdale et al., 2022), and one both hip and knee joints (Sukhonthamarn et al., 2020). Infection was defined in two studies according to the MSIS 2011 definition (Workgroup and Society, 2011), in one study according to IC 2013 (Parvizi and Gehrke, 2014) and in one according to IC 2018 (Goswami et al., 2018), and the remaining two used positive cultures and purulence. Time for diagnosis was determined at 3 weeks in one study and 6 weeks in two studies, while three studies determined data at 6 weeks and 12 weeks. Table 1 shows more general characteristics of the primary studies.

**Table 1:** General characteristics of all included primary studies. THA: total hip arthroplasty; UKA: unicompartmental knee arthroplasty.

Author	Year of publ.	Infected patients/total	Joint	Prosthesis	Demographics	Infection definition	Time to diagnosis	Leucocyte cutoff value	PMN cutoff value	Reporting funds
Bedair et al. (2011)	2010	19/146	Knee	Primary TKA	Mean age 66.1 85 women (58 %) Mean time index surgery to aspiration: (infected 20.8 d, non-infected 15.9 d)	Positive cultures, grossly purulent material	6 weeks	10 700 and 27 800	>89 %	No funds mentioned, Mark Coventry award
Yi et al. (2014)	2013	36/73	Hip	Primary THA	Mean age 60 34 women (47 %) Mean time index surgery to reoperation: 23 d	Modified criteria of Workgroup and Society (2011). (1) Sinus tract (2) Pathogen in two or more cultures 3.4 of 6 minor criteria (IC 2013)	6 weeks	12 800	>89 %	No funds mentioned, Frank Stinchfield award
Kim et al. (2017)	2017	12/197	Knee	Primary TKA and UKA	Mean age: 69 163 women Mean time index surgery to aspirate: (infected 11.4 d, non-infected 10.9 d)	Positive cultures, Purulent fluid	3 weeks	11 200 and 16 000	>88 % and >93 %	Not mentioned
Sukhonthamarn et al. (2020)	2020	123/197	Knee or hip	Primary TKA or THA	Mean age; 67.5 infected, 69.9 non-infected Mean time index surgery to aspirate: infected 35.1 d, non-infected 15.5 d	IC 2018	12 weeks and 6 weeks	6130 (90 d = 12 weeks) 8145 (45 d = 6 weeks)	>79.5 % >84.5 %	No external funding or grants
Uvodich et al. (2021)	2021	22/170	Knee	Primary TKA	Mean age: infected 63. Non-infected 67 85 women Mean time index surgery to aspirate: infected <6 weeks 24 d, 6–12 weeks 64 d; non-infected <6 weeks 19 d, 6–12 weeks 61 d	Major criteria MSIS 2011	6 weeks and 12 weeks	6 weeks: 8676 6–12 weeks: 1983	6 weeks: >88 % 6–12 weeks: >76 %	No external funding or grants
Dugdale et al. (2022)	2021	15/43	Hip	Primary THA	Median age: infected 66, non-infected 63 Median time index surgery to aspirate: infected <6 weeks 22 d, 6–12 weeks 58 d; non-infected <6 weeks 25 d, 6–12 weeks 65 d	Major criteria MSIS 2011	6 weeks and 12 weeks	6 weeks: 4390 6–12 weeks: 26 995	6 weeks: >74 % 6–12 weeks: >93 %	Not mentioned

Cutoff values for WBC count range from 1983 to 27 800 cells, with a median of 10 000 cells. The 3-, 6- and 12-week median was 13 600, 9338 and 6130 respectively. For the PMN percentage, cutoff values range from 70 % to 93 %, with a median of 88 %. The 3-, 6- and 12-week median was 90.5 %, 88 % and 79.5 % respectively.

**Table 2:** DOR meta-analysis for synovial leucocyte count and PMN percentage. REM: random effect model; d.f.: degrees of freedom.

Summary diagnostic odds ratio (random effects model) for leucocytes			
Study	DOR	(95 % conf. interval)	% weight
Bedair et al. (2011)	189.82	23.094–1560.2	8.67
Bedair et al. (2011)	672	65.896–6853	7.68
Yi et al. (2014)	541.67	28.089–10 445.4	5.48
Kim et al. (2017)	1971	90.018–431 556.2	5.15
Kim et al. (2017)	1107	53.606–22 860.1	5.3
Sukhonthamarn et al. (2020)	48.126	20.345–113.84	17.23
Sukhonthamarn et al. (2020)	52.221	21.353–127.71	16.96
Uvodich et al. (2021)	44.375	8.926–22.62	11.57
Uvodich et al. (2021)	22.667	4.535–113.3	11.53
Dugdale et al. (2022)	175.50	14.551–2116.7	7.01
Dugdale et al. (2022)	1767	33.401–93 478.2	3.44
(REM) pooled DOR	123.61	55.388–275.88	
Heterogeneity chi-squared: 20.36 (d.f. = 10), p=0.026			
Inconsistency (I-squared): 50.9 %			
Estimate of between-study variance (Tau-squared): 0.781			
Number of studies: 11			
Summary diagnostic odds ratio (random effects model) for PMN			
Study	DOR	(95 % conf. interval)	% weight
Bedair et al. (2011)	12.034	3.314–43.695	10.8
Yi et al. (2014)	35.214	9.364–132.43	10.35
Kim et al. (2017)	51.661	3.022–883.3	2.65
Kim et al. (2017)	14.31	4.096–49.997	11.33
Sukhonthamarn et al. (2020)	33.213	12.143–90.840	15.71
Sukhonthamarn et al. (2020)	16.006	7.378–34.725	22.05
Uvodich et al. (2021)	7.029	1.998–24.719	11.24
Uvodich et al. (2021)	17.333	3.501–85.813	7.55
Dugdale et al. (2022)	14.444	2.682–77.797	6.91
Dugdale et al. (2022)	1829	34.593–96 702.4	1.39
(REM) pooled DOR	18.712	11.644–30.073	
Heterogeneity chi-squared: 11.15 (d.f. = 9), p=0.26			
Inconsistency ( I -squared): 19.3 %			
Estimate of between-study variance (Tau-squared): 0.1095			
Number of studies: 10			

**Figure 3:**
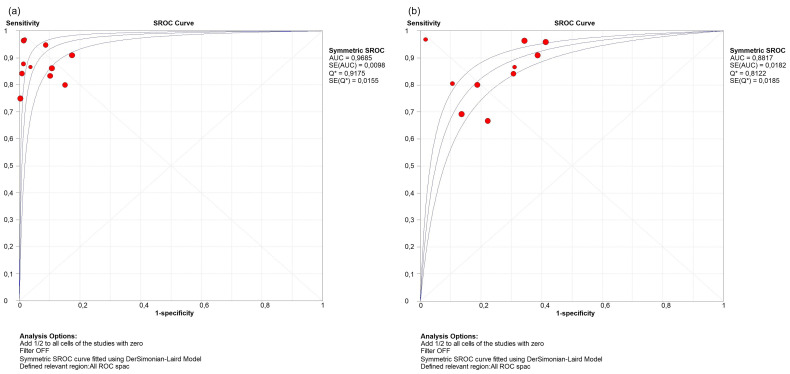
sROC for synovial leucocyte count **(a)** and PMN percentage **(b)**.

**Figure 4:**
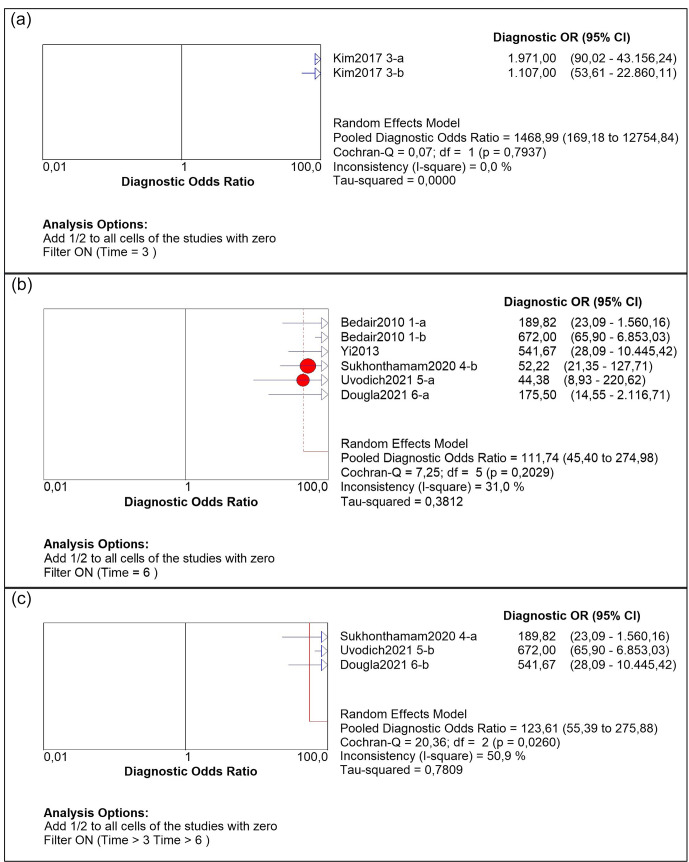
DOR subgroup analysis for synovial leucocyte count: **(a)** 3 weeks; **(b)** 6 weeks; **(c)** 12 weeks.

**Figure 5:**
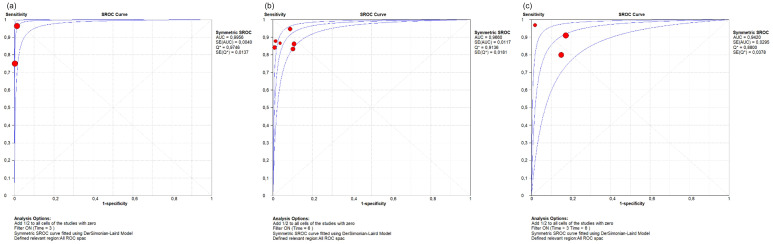
sROC subgroup analysis for synovial leucocyte count: **(a)** 3 weeks; **(b)** 6 weeks; **(c)** 12 weeks.

### 3.4 Meta-analysis

The DOR and sROC for WBC count and the PMN percentage were calculated using data extracted from each study. WBC count showed a summary DOR of 123.61 (95 % CI: 55.38–275.88), an sROC with an AUC of 0.96 (SE: 0.009) and a Q index of 0.917. The PMN percentage showed a summary DOR of 18.71 (95 % CI: 11.64–30.07), an sROC with an AUC of 0.88 (SE: 0.018) and a Q index of 0.812 (Table 2 and Fig. 3).

We performed a subgroup analysis by time of diagnosis at 3, 6 and 12 weeks. For WBC count, the DOR was 1468.99 (95 % CI 169.18–12 754.84), 111.74 (95 % IC 45.4–274.98) and 123.61 (95 % IC 55.30–275.88) respectively (Fig. 4). For the PMN percentage, the DOR was 18.71 (95 % CI 11.64–30.07) in all subgroups. As for the sROC AUC and Q index for WBC count, the figures were as follows: an AUC of 0.9956 and a Q of 0.976, an AUC of 0.9660 and a Q of 0.9136, and an AUC of 0.9420 and a Q of 0.88 respectively (Fig. 5). For the PMN percentage, the sROC showed an AUC of 0.88 and a Q index of 0.812 in all subgroups.

Heterogeneity ( $I^{2}$ ) in sensitivity and specificity for WBC count and the PMN percentage were 14.6 % and 88.6 % and 64.9 % and 86.6 % respectively. As sensitivity and specificity can create a threshold effect (de Sousa et al., 2009), we also calculated heterogeneity based on the DOR, finding $I^{2}$ 50.9 % for WBC and 19.3 % for the PMN percentage, which represents moderate heterogeneity.

## 4 Discussion

This study showed that both synovial WBC count and PMN percentage are good markers for diagnosis of acute postoperative PJI. Furthermore, our findings suggest that leucocyte counts were more powerful in diagnosing infections of this type, with a DOR of 123.6 for leucocytes vs. 18.7 for the PMN percentage and an AUC in the sROC of 0.96 for leucocytes vs. 0.88 for the PMN percentage.

There is currently no gold-standard test for PJI diagnosis. Therefore, diagnosis of the condition relies on consensus, in which different tests are performed (Goswami et al., 2018; Trampuz and Zimmerli, 2005; Signore et al., 2019). In acute postoperative PJI, diagnosis is even more difficult because the existing literature is oriented more toward chronic PJI, and no clear cutoff values have been established for acute vs. chronic infection. Acute postoperative diagnosis is variously defined as 3, 6 or 12 weeks, with no consensus as to which should be used (Zimmerli et al., 2004; Trampuz and Zimmerli, 2005; Osmon et al., 2013). In this study, we divided our subgroup analysis into three groups (3, 6 and 12 weeks) and found no differences in DOR or sROC analysis between 3-, 6- or 12-week groups for the PMN percentage, at 18.7 and 0.88 respectively. When comparing the groups for synovial WBC count, in the 3-week group the DOR and sROC were demonstrated as more powerful. However, we believe that these results should not be regarded as definitive because there is only one study, by Kim et al. (2017), with two different cutoff values for this period of analysis. Consequently, our interpretation is that the group with a higher DOR was the 12-week group, while for sROC it was the 6-week group. These discrepancies may be explained by a large confidence interval for the DOR.

One of the limitations of this SR is the small number of primary studies included, due to very strict inclusion criteria. Therefore, publication bias was not calculated because tests for funnel plot asymmetry should not be used when there are fewer than 10 primary studies in the MA, as recommended by Sterne et al. (2011). Overall, the risk of bias was considered high though they provided good scores for applicability. The risk of bias was always considered high on patient selection and the reference standard domain because the studies under consideration were retrospective. Acute postoperative PJI is a rare condition, making retrospective studies more useful. As for the reference standard domain, the definition of PJI has changed over years, but consensus has always included synovial WBC count and PMN for PJI diagnosis. It is obvious that when studying WBC and PMN percentage diagnostic accuracy, these values should not be considered when defining PJI. Another limitation, related to the retrospective nature of the primary studies, is the moderate heterogeneity between studies. Randomized control trials (RTCs) could control this heterogeneity, though considering that this is a rare condition and that RTCs are not possible for this type of condition due to ethical reasons such as aspiration during the postoperative period of joints without any clinical suspicion of infection, the only study possible is a retrospective study.

In the study by Christensen et al. (2013), the natural progression of synovial WBC count and PMN percentage was studied. The study demonstrated that chronic PJI cutoff values for these biomarkers were not useful during the first 6 weeks after TKA (total knee arthroplasty), as the natural progression demonstrated higher concentrations than those used for the diagnosis of chronic PJI. Although, with the results found in this SR, we cannot determine the cutoff values for synovial WBC count and PMN percentage, considering the literature reviewed and our findings, we believe that the real threshold may be 10 000 cells µL or higher for WBC count and 85 % or higher for the PMN percentage.

Although useful, these measures are not 100 % accurate; when a doubt exists, cultures can aid in diagnosis. However, relying completely on cultures can delay treatment. Therefore, studying other biomarkers such as synovial glucose level could help clinicians reach a diagnosis. Synovial glucose is a well-known parameter for diagnosis of septic native joint infection, but it has not been studied in acute postoperative PJI, and its natural cycle has not yet been established.

In conclusion, both synovial WBC count and PMN percentage are useful in the diagnosis of acute postoperative PJI, with WBC being the more accurate of the two.

## Supplement

10.5194/jbji-9-17-2024-supplement The supplement related to this article is available online at: https://doi.org/10.5194/jbji-9-17-2024-supplement.

## Data Availability

Data used in this article are available at https://doi.org/10.17605/OSF.IO/9P6C7 (Sabater-Martos et al., 2024).
