# Supplementary material for: Diagnostic cutoff values of synovial fluid biomarkers for acute postoperative prosthetic joint infection: a systematic review and meta-analysis
Source: J Bone Jt Infect. 2024 Jan 29;9(1):17–26. doi: 10.5194/jbji-9-17-2024 (PMC11002915; doi:10.5194/jbji-9-17-2024)
Supplement: The supplement related to this article is available online at: https://doi.org/10.5194/jbji-9-17-2024-supplement. [file jbji-9-17-supplement.pdf]

Supplement of J. Bone Joint Infect., 9, 17–26, 2024
https://doi.org/10.5194/jbji-9-17-2024-supplement
© Author(s) 2024. CC BY 4.0 License.

Supplement of

Diagnostic cutoff values of synovial fluid biomarkers for acute postoperative prosthetic joint infection: a systematic review and meta-analysis

Marta Sabater-Martos et al.

Correspondence to: Marta Sabater-Martos (msabater@clinic.cat)

The copyright of individual parts of the supplement might differ from the article licence.

Table S1: Search strategy for MedLine and Embase

Search strategy for MedLine/PubMed—29/12/2021
((("Arthroplasty, Replacement, Knee"[Mesh]) OR (prosthetic joint*[tiab]) OR (periprosthetic joint*[tiab]) OR (joint arthoplast*[tiab]) OR (joint arthroplast*[tiab]) OR (joint replace*[tiab]) OR (TKA*[tiab]) OR (arthroplast* [ti]) OR (arthoplast*[ti]) OR (periprosthetic[ti]) OR (knee*[ti])) AND (("Prosthesis-Related Infections"[Mesh]) OR (acute infection*[ti]) OR (PJI* [tiab]))) AND ((sensitivity[tiab]) OR (specificity[tiab]) OR (synovial count* [tiab]) OR (leucocyt* [tiab]) OR (PMN [tiab]) OR (neutrohpil [tiab]) OR (synovial [tiab]))

Search strategy for Embase—29/12/2021
('replacement arthroplasty'/exp OR ((prosthetic NEAR/2 joint*):ab,ti) OR ((periprosthetic NEAR/2 joint*):ab,ti) OR ((arth*oplast* NEAR/2 joint*):ab,ti) OR tka*:ab,ti OR tja*:ab,ti OR arth*oplast*:ti OR periprosthetic:ti OR joint:ti OR joints:ti) AND ('prosthesis infection'/exp OR ((acute* NEAR/2 infection*):ti) OR pji*:ti) AND (specificity:ab,ti OR sensitivity:ab,ti OR ((synovial NEAR/2 count):ab,ti) OR ((synovial NEAR/2 fluid):ab,ti) OR synovial:ab,ti OR leucocyte*:ab,ti OR pmn*:ab,ti OR neutrophil*:ab,ti)

Table S2: Characteristics of the excluded SR after full text reading

	Author and publication year	Title	Reason for exclusion
1	Pagliaccetti J. et al. 2021	Variability and Interpretation of Synovial Cell Count and Differential: A Perspective in Hip and Knee Arthroplasty	Wrong study design
2	Fernández-Sampedro, M et al. 2017	Accuracy of different diagnostic tests for early, delayed and late prosthetic joint infection	Wrong Outcome
3	Zhang, Chao-Fan et al. 2020	Debridement, Antibiotics, and Implant Retention for Acute Periprosthetic Joint Infection	Wrong Outcome
4	Yu, Bao-Zhan et al. 2020	Neutrophil to lymphocyte ratio as a predictor for diagnosis of early Periprosthetic joint infection	Wrong Outcome
5	Li, Hao et al. 2021	The concordance between preoperative aspiration and intraoperative synovial fluid culture results: intraoperative synovial fluid re-cultures are necessary whether the preoperative aspiration culture is positive or not	Wrong Outcome
6	Xu C. et al. 2019	Reevaluating Current Cutoffs for Acute Periprosthetic Joint Infection: Current Thresholds Are Insensitive	Only reporting sensitivity
7	Mason, J Bohannon et al. 2003	The value of white blood cell counts before revision total knee arthroplasty	Wrong population

8	den Bekerom, Michel P J et al. 2006	The value of pre-operative aspiration in the diagnosis of an infected prosthetic knee: a retrospective study and review of literature	Wrong population
9	Baré et al. 2006	Preoperative evaluations in revision total knee arthroplasty	Wrong population
10	Ghanem E. et al. 2008	Cell count and differential of aspirated fluid in the diagnosis of infection at the site of total knee arthroplasty	Wrong population
11	Schinsky, Mark F et al. 2008	Perioperative testing for joint infection in patients undergoing revision total hip arthroplasty	Wrong population
12	Lee, Su Chan et al. 2010	Analysis of synovial fluid in culture-negative samples of suspicious periprosthetic infections	Wrong population
13	Society of Unicondylar Research and Continuing Education. 2012	Diagnosis of periprosthetic joint infection after unicompartmental knee arthroplasty	Wrong population
14	Zmistowski, Benjamin et al. 2012	Periprosthetic joint infection diagnosis: a complete understanding of white blood cell count and differential	Wrong population
15	Dinneen, A et al. 2013	Synovial fluid white cell and differential count in the diagnosis or exclusion of prosthetic joint infection	Wrong population
16	Christensen, Christian P. et al. 2013	The natural progression of synovial fluid white blood-cell counts and the percentage of polymorphonuclear cells after primary total knee arthroplasty: a multicenter study	Wrong population
17	Lenski, Markus et al. 2014	Synovial IL-6 as inflammatory marker in periprosthetic joint infections	Wrong population
18	Claassen, Leif et al. 2014	Preoperative diagnostic for periprosthetic joint infection prior to total knee revision arthroplasty	Wrong population
19	Lenski Markus et al. 2015	Diagnostic potential of inflammatory markers in septic arthritis and periprosthetic joint infections: a clinical study with 719 patients	Wrong population
20	Shafafy, R et al. 2015	Use of leucocyte esterase reagent strips in the diagnosis or exclusion of prosthetic joint infection	Wrong population
21	Sousa, R et al. 2017	Improving the accuracy of synovial fluid analysis in the diagnosis of prosthetic joint infection with simple and inexpensive biomarkers	Wrong population
22	Gallo, Jiri et al. 2017	Excellent AUC for joint fluid cytology in the detection/exclusion of hip and knee prosthetic joint infection	Wrong population
23	Shahi, Alisina et al. 2017	Diagnosing Periprosthetic Joint Infection: And the Winner Is?	Wrong population
24	De Vecchi, Elena et al. 2018	Alpha defensin, leukocyte esterase, C-reactive protein, and leukocyte count in synovial fluid for pre-operative diagnosis of periprosthetic infection	Wrong population
25	Parvizi, Javad et al. 2018	The 2018 Definition of Periprosthetic Hip and Knee Infection: An Evidence-Based and Validated Criteria	Wrong population and outcome
26	Strahm, Carol et al 2018	Accuracy of Synovial Leukocyte and Polymorphonuclear Cell Count in Patients with Shoulder Prosthetic Joint Infection	Wrong population
27	Klim, S M et al. 2018	Fibrinogen - A Practical and Cost Efficient Biomarker for Detecting Periprosthetic Joint Infection	Wrong population

28	Zahar, Akos et al. 2018	How Reliable Is the Cell Count Analysis in the Diagnosis of Prosthetic Joint Infection?	Wrong population
29	Ding, Benjamin Tk et al. 2019	Accuracy of the α-defensin lateral flow assay for diagnosing periprosthetic joint infection in Asians	Wrong population
30	Tahta, Mesut et al. 2019	Does inflammatory joint diseases affect the accuracy of infection biomarkers in patients with periprosthetic joint infections? A prospective comparative reliability study	Wrong population
31	Shahi, Alisina et al. 2019	The Leukocyte Esterase Test for Periprosthetic Joint Infection Is Not Affected by Prior Antibiotic Administration	Wrong population
32	Lazarides, Alexander L et al. 2019	Traditional Laboratory Markers Hold Low Diagnostic Utility for Immunosuppressed Patients With Periprosthetic Joint Infections	Wrong population
33	Yermak, Katsiaryna et al. 2019	Performance of synovial fluid D-lactate for the diagnosis of periprosthetic joint infection: A prospective observational study	Wrong population
34	Qin, Leilei et al. 2020	Evaluation of synovial fluid neutrophil CD64 index as a screening biomarker of prosthetic joint infection	Wrong population
35	Chu, Lei et al. 2020	The combinations of multiple factors to improve the diagnostic sensitivity and specificity after artificial joint infection	Wrong population
36	Bäcker, Henrik C et al. 2020	Increased Synovial Inflammatory Markers in Aseptic Total Hip Arthroplasty Dislocation	Wrong population and outcome
37	Deirmengian, Carl A et al. 2020	False-Positive Automated Synovial Fluid White Blood Cell Counting Is a Concern for Both Hip and Knee Arthroplasty Aspirates	Wrong population
38	Zhao, Guanglei et al 2020	Predictive values of the postoperative neutrophil-to-lymphocyte ratio, platelet-to-lymphocyte ratio, and lymphocyte-to-monocyte ratio for the diagnosis of early periprosthetic joint infections: a preliminary study	Wrong population
39	Mihalič, René et al. 2020	Synovial fluid interleukin-6 is not superior to cell count and differential in the detection of periprosthetic joint infection	Wrong population
40	Sharma, Katyayini et al. 2020	Comparative analysis of 23 synovial fluid biomarkers for hip and knee periprosthetic joint infection detection.	Wrong population
41	Tirumala, Venkatsaiakhil et al. 2021	Diagnostic Utility of Platelet Count/Lymphocyte Count Ratio and Platelet Count/Mean Platelet Volume Ratio in Periprosthetic Joint Infection Following Total Knee Arthroplasty.	Wrong population
42	Fink, Bernd et al. 2021	The Graphical Representation of Cell Count Representation: A New Procedure for the Diagnosis of Periprosthetic Joint Infections.	Wrong population
43	Ivy, Morgan I et al. 2021	Synovial fluid α defensin has comparable accuracy to synovial fluid white blood cell count and polymorphonuclear percentage for periprosthetic joint infection diagnosis	Wrong population
44	Salar, Omer et al. 2021	Diagnosis of knee prosthetic joint infection; aspiration and biopsy	Wrong population

<u>Table S3: </u>Additional information about QUADAS-2 application

Paper 1: Bedair et al. 2011

<u>Domain 1 Patient selection</u>: Describe included patients (prior testing, presentation, intended use of index test and setting)

Questions	Answer (Yes, No or Unclear)	Motive
1.1 Was a consecutive or random sample of patients enrolled?	No	Retrospective
1.2 Was a case-control design avoided?	Unclear	
1.3 Did the study avoid inappropriate exclusions?	yes	
RISK OF BIAS: Could the selection of patients have introduced bias? (High, Low or Unclear)	HIGH	
APPLICABILITY: Are there concerns that the included patients do not match the review question? (High, Low or Unclear)	LOW	

<u>Domain 2 Index test</u>: Describe the index test and how it was conducted and interpreted

Questions	Answer (Yes, No or Unclear)	Motive
2.1 Were the index test results interpreted without knowledge of the results of the reference standard?	Yes	
2.2 If a threshold was used, was it pre-specified??	Yes	
RISK OF BIAS: Could the conduct or interpretation of the index test have introduced bias? (High, Low or Unclear)	LOW	
APPLICABILITY: Are there concerns that the index test, its conduct, or interpretation differ from the review question?? (High, Low or Unclear)	LOW	

<u>Domain 3 Reference Standard:</u> Describe the reference standard and how it was conducted and interpreted:

Questions	Answer (Yes, No or Unclear)	Motive
3.1 Is the reference standard likely to correctly classify the target condition?	No	Cultures may be negative
3.2 Were the reference standard results interpreted without knowledge of the results of the index test?	Yes	
RISK OF BIAS:	HIGH	

Could the reference standard, its conduct, or its interpretation have introduced bias? (High, Low or Unclear)		
APPLICABILITY:<br>Are there concerns that the target condition as defined by the reference standard does not match the review question? (High, Low or Unclear)	LOW	

<u>Domain 4 Flow and Timing</u>: Describe any patients who did not receive the index test(s) and/or reference standard or who were excluded from the 2x2 table (refer to flow diagram): Describe the time interval and any interventions between index test(s) and reference standard:

Questions	Answer (Yes, No or Unclear)	Motive
4.1 Was there an appropriate interval between index test(s) and reference standard?	Unclear	Not mention when day did the aspiration and the cultures
4.2 Did all patients receive a reference standard??	yes	
4.3 Did all patients receive the same reference standard??	yes	
4.4 Were all patients included in the analysis?	Yes	
RISK OF BIAS:<br>Could the patient flow have introduced bias? (High, Low or Unclear)	LOW	

Paper 2: Yi et al. 2014

Domain 1 Patient selection: Describe included patients (prior testing, presentation, intended use of index test and setting)

Questions	Answer (Yes, No or Unclear)	Motive
1.1 Was a consecutive or random sample of patients enrolled?	No	Retrospective
1.2 Was a case-control design avoided?	Unclear	
1.3 Did the study avoid inappropriate exclusions?	No	They only include patients undergoing surgery
RISK OF BIAS:<br>Could the selection of patients have introduced bias? (High, Low or Unclear)	HIGH	
APPLICABILITY:<br>Are there concerns that the included patients do not match the review question? (High, Low or Unclear)	UNCLEAR	

Domain 2 Index test: Describe the index test and how it was conducted and interpreted

Questions	Answer (Yes, No or Unclear)	Motive
2.1 Were the index test results interpreted without knowledge of the results of the reference standard?	Yes	
2.2 If a threshold was used, was it pre-specified??	-	Non used
RISK OF BIAS:<br>Could the conduct or interpretation of the index test have introduced bias? (High, Low or Unclear)	LOW	
APPLICABILITY:<br>Are there concerns that the index test, its conduct, or interpretation differ from the review question?? (High, Low or Unclear)	LOW	

Domain 3 Reference Standard: Describe the reference standard and how it was conducted and interpreted:

Questions	Answer (Yes, No or Unclear)	Motive
3.1 Is the reference standard likely to correctly classify the target condition?	No	Cultures may be negative
3.2 Were the reference standard results interpreted without knowledge of the results of the index test?	Unclear	retrospective
RISK OF BIAS:<br>Could the reference standard, its conduct, or its interpretation	HIGH	

have introduced bias? (High, Low or Unclear)		
APPLICABILITY:<br>Are there concerns that the target condition as defined by the reference standard does not match the review question? (High, Low or Unclear)	LOW	

<u>Domain 4 Flow and Timing</u>: Describe any patients who did not receive the index test(s) and/or reference standard or who were excluded from the 2x2 table (refer to flow diagram): Describe the time interval and any interventions between index test(s) and reference standard:

Questions	Answer (Yes, No or Unclear)	Motive
4.1 Was there an appropriate interval between index test(s) and reference standard?	Unclear	
4.2 Did all patients receive a reference standard??	Yes	
4.3 Did all patients receive the same reference standard??	Yes	
4.4 Were all patients included in the analysis?	Yes	
RISK OF BIAS:<br>Could the patient flow have introduced bias? (High, Low or Unclear)	LOW	

Paper 3: Kim et al. 2017

Domain 1 Patient selection: Describe included patients (prior testing, presentation, intended use of index test and setting)

Questions	Answer (Yes, No or Unclear)	Motive
1.1 Was a consecutive or random sample of patients enrolled?	No	retrospective
1.2 Was a case-control design avoided?	Unclear	
1.3 Did the study avoid inappropriate exclusions?	Yes	They exclude patients but are justified
RISK OF BIAS: Could the selection of patients have introduced bias? (High, Low or Unclear)	HIGH	
APPLICABILITY: Are there concerns that the included patients do not match the review question? (High, Low or Unclear)	LOW	

Domain 2 Index test: Describe the index test and how it was conducted and interpreted

Questions	Answer (Yes, No or Unclear)	Motive
2.1 Were the index test results interpreted without knowledge of the results of the reference standard?	Yes	
2.2 If a threshold was used, was it pre-specified??	Yes	
RISK OF BIAS: Could the conduct or interpretation of the index test have introduced bias? (High, Low or Unclear)	LOW	
APPLICABILITY: Are there concerns that the index test, its conduct, or interpretation differ from the review question?? (High, Low or Unclear)	LOW	

Domain 3 Reference Standard: Describe the reference standard and how it was conducted and interpreted:

Questions	Answer (Yes, No or Unclear)	Motive
3.1 Is the reference standard likely to correctly classify the target condition?	No	Negative cultures
3.2 Were the reference standard results interpreted without knowledge of the results of the index test?	Yes	
RISK OF BIAS: Could the reference standard, its conduct, or its interpretation	HIGH	

have introduced bias? (High, Low or Unclear)		
APPLICABILITY:<br>Are there concerns that the target condition as defined by the reference standard does not match the review question? (High, Low or Unclear)	LOW	

Domain 4 Flow and Timing: Describe any patients who did not receive the index test(s) and/or reference standard or who were excluded from the 2x2 table (refer to flow diagram): Describe the time interval and any interventions between index test(s) and reference standard:

Questions	Answer (Yes, No or Unclear)	Motive
4.1 Was there an appropriate interval between index test(s) and reference standard?	Unclear	
4.2 Did all patients receive a reference standard??	Yes	
4.3 Did all patients receive the same reference standard??	Yes	
4.4 Were all patients included in the analysis?	No	Exclusions are justified
RISK OF BIAS:<br>Could the patient flow have introduced bias? (High, Low or Unclear)	Unclear	

Paper 4: Xu et al. 2019

Domain 1 Patient selection: Describe included patients (prior testing, presentation, intended use of index test and setting)

Questions	Answer (Yes, No or Unclear)	Motive
1.1 Was a consecutive or random sample of patients enrolled?	No	Not mentioned but retrospective
1.2 Was a case-control design avoided?	yes	
1.3 Did the study avoid inappropriate exclusions?	No	
RISK OF BIAS: Could the selection of patients have introduced bias? (High, Low or Unclear)	HIGH	
APPLICABILITY: Are there concerns that the included patients do not match the review question? (High, Low or Unclear)	UNCLEAR	

Domain 2 Index test: Describe the index test and how it was conducted and interpreted

Questions	Answer (Yes, No or Unclear)	Motive
2.1 Were the index test results interpreted without knowledge of the results of the reference standard?	Yes	
2.2 If a threshold was used, was it pre-specified??	Unclear	
RISK OF BIAS: Could the conduct or interpretation of the index test have introduced bias? (High, Low or Unclear)	UNCLEAR	
APPLICABILITY: Are there concerns that the index test, its conduct, or interpretation differ from the review question? (High, Low or Unclear)	LOW	

Domain 3 Reference Standard: Describe the reference standard and how it was conducted and interpreted:

Questions	Answer (Yes, No or Unclear)	Motive
3.1 Is the reference standard likely to correctly classify the target condition?	No	
3.2 Were the reference standard results interpreted without knowledge of the results of the index test?	Yes	
RISK OF BIAS: Could the reference standard, its conduct, or its interpretation	UNCLEAR	

have introduced bias? (High, Low or Unclear)		
APPLICABILITY:<br>Are there concerns that the target condition as defined by the reference standard does not match the review question? (High, Low or Unclear)	LOW	

Domain 4 Flow and Timing: Describe any patients who did not receive the index test(s) and/or reference standard or who were excluded from the 2x2 table (refer to flow diagram): Describe the time interval and any interventions between index test(s) and reference standard:

Questions	Answer (Yes, No or Unclear)	Motive
4.1 Was there an appropriate interval between index test(s) and reference standard?	Unclear	Not mentioned
4.2 Did all patients receive a reference standard??	Unclear	
4.3 Did all patients receive the same reference standard??	Unclear	
4.4 Were all patients included in the analysis?	No	
RISK OF BIAS:<br>Could the patient flow have introduced bias? (High, Low or Unclear)	HIGH	

Paper 5: Sukhonthamarn et al. 2020

Domain 1 Patient selection: Describe included patients (prior testing, presentation, intended use of index test and setting)

Questions	Answer (Yes, No or Unclear)	Motive
1.1 Was a consecutive or random sample of patients enrolled?	No	Retrospective
1.2 Was a case-control design avoided?	Unclear	
1.3 Did the study avoid inappropriate exclusions?	Yes	
RISK OF BIAS: Could the selection of patients have introduced bias? (High, Low or Unclear)	HIGH	
APPLICABILITY: Are there concerns that the included patients do not match the review question? (High, Low or Unclear)	LOW	

Domain 2 Index test: Describe the index test and how it was conducted and interpreted

Questions	Answer (Yes, No or Unclear)	Motive
2.1 Were the index test results interpreted without knowledge of the results of the reference standard?	Yes	
2.2 If a threshold was used, was it pre-specified??	Unclear	Not mentioned
RISK OF BIAS: Could the conduct or interpretation of the index test have introduced bias? (High, Low or Unclear)	LOW	
APPLICABILITY: Are there concerns that the index test, its conduct, or interpretation differ from the review question?? (High, Low or Unclear)	LOW	

Domain 3 Reference Standard: Describe the reference standard and how it was conducted and interpreted:

Questions	Answer (Yes, No or Unclear)	Motive
3.1 Is the reference standard likely to correctly classify the target condition?	No	Cultures may be negative
3.2 Were the reference standard results interpreted without knowledge of the results of the index test?	Yes	
RISK OF BIAS: Could the reference standard, its conduct, or its interpretation	HIGH	

have introduced bias? (High, Low or Unclear)		
APPLICABILITY:<br>Are there concerns that the target condition as defined by the reference standard does not match the review question? (High, Low or Unclear)	LOW	

Domain 4 Flow and Timing: Describe any patients who did not receive the index test(s) and/or reference standard or who were excluded from the 2x2 table (refer to flow diagram): Describe the time interval and any interventions between index test(s) and reference standard:

Questions	Answer (Yes, No or Unclear)	Motive
4.1 Was there an appropriate interval between index test(s) and reference standard?	Unclear	Not mentioned
4.2 Did all patients receive a reference standard??	Yes	
4.3 Did all patients receive the same reference standard??	Yes	
4.4 Were all patients included in the analysis?	Yes	
RISK OF BIAS:<br>Could the patient flow have introduced bias? (High, Low or Unclear)	LOW	

Paper 6: Uvodich et al. 2021

Domain 1 Patient selection: Describe included patients (prior testing, presentation, intended use of index test and setting)

Questions	Answer (Yes, No or Unclear)	Motive
1.1 Was a consecutive or random sample of patients enrolled?	No	Retrospective
1.2 Was a case-control design avoided?	Unclear	
1.3 Did the study avoid inappropriate exclusions?	Yes	
RISK OF BIAS: Could the selection of patients have introduced bias? (High, Low or Unclear)	HIGH	
APPLICABILITY: Are there concerns that the included patients do not match the review question? (High, Low or Unclear)	LOW	

Domain 2 Index test: Describe the index test and how it was conducted and interpreted

Questions	Answer (Yes, No or Unclear)	Motive
2.1 Were the index test results interpreted without knowledge of the results of the reference standard?	Yes	
2.2 If a threshold was used, was it pre-specified??	Unclear	Not specified
RISK OF BIAS: Could the conduct or interpretation of the index test have introduced bias? ? (High, Low or Unclear)	LOW	
APPLICABILITY: Are there concerns that the index test, its conduct, or interpretation differ from the review question?? (High, Low or Unclear)	LOW	

Domain 3 Reference Standard: Describe the reference standard and how it was conducted and interpreted:

Questions	Answer (Yes, No or Unclear)	Motive
3.1 Is the reference standard likely to correctly classify the target condition?	No	Major criteria, Cultures may be negative
3.2 Were the reference standard results interpreted without knowledge of the results of the index test?	Yes	
RISK OF BIAS: Could the reference standard, its conduct, or its interpretation	HIGH	

have introduced bias? (High, Low or Unclear)		
APPLICABILITY:<br>Are there concerns that the target condition as defined by the reference standard does not match the review question? (High, Low or Unclear)	LOW	

Domain 4 Flow and Timing: Describe any patients who did not receive the index test(s) and/or reference standard or who were excluded from the 2x2 table (refer to flow diagram): Describe the time interval and any interventions between index test(s) and reference standard:

Questions	Answer (Yes, No or Unclear)	Motive
4.1 Was there an appropriate interval between index test(s) and reference standard?	Unclear	Not mentioned
4.2 Did all patients receive a reference standard??	Yes	
4.3 Did all patients receive the same reference standard??	Yes	
4.4 Were all patients included in the analysis?	Yes	
RISK OF BIAS:<br>Could the patient flow have introduced bias? (High, Low or Unclear)	LOW	

Paper 7: Dugdale et al.2021

Domain 1 Patient selection: Describe included patients (prior testing, presentation, intended use of index test and setting)

Questions	Answer (Yes, No or Unclear)	Motive
1.1 Was a consecutive or random sample of patients enrolled?	No	Retrospective
1.2 Was a case-control design avoided?	Unclear	
1.3 Did the study avoid inappropriate exclusions?	Yes	
RISK OF BIAS: Could the selection of patients have introduced bias? (High, Low or Unclear)	HIGH	
APPLICABILITY: Are there concerns that the included patients do not match the review question? (High, Low or Unclear)	LOW	

Domain 2 Index test: Describe the index test and how it was conducted and interpreted

Questions	Answer (Yes, No or Unclear)	Motive
2.1 Were the index test results interpreted without knowledge of the results of the reference standard?	Yes	
2.2 If a threshold was used, was it pre-specified??	Unclear	Not specified
RISK OF BIAS: Could the conduct or interpretation of the index test have introduced bias? ? (High, Low or Unclear)	LOW	
APPLICABILITY: Are there concerns that the index test, its conduct, or interpretation differ from the review question?? (High, Low or Unclear)	LOW	

Domain 3 Reference Standard: Describe the reference standard and how it was conducted and interpreted:

Questions	Answer (Yes, No or Unclear)	Motive
3.1 Is the reference standard likely to correctly classify the target condition?	No	Major criteria, Cultures may be negative
3.2 Were the reference standard results interpreted without knowledge of the results of the index test?	Yes	
RISK OF BIAS: Could the reference standard, its conduct, or its interpretation	HIGH	

have introduced bias? (High, Low or Unclear)		
APPLICABILITY:<br>Are there concerns that the target condition as defined by the reference standard does not match the review question? (High, Low or Unclear)	LOW	

Domain 4 Flow and Timing: Describe any patients who did not receive the index test(s) and/or reference standard or who were excluded from the 2x2 table (refer to flow diagram): Describe the time interval and any interventions between index test(s) and reference standard:

Questions	Answer (Yes, No or Unclear)	Motive
4.1 Was there an appropriate interval between index test(s) and reference standard?	Unclear	Not mentioned
4.2 Did all patients receive a reference standard??	Yes	
4.3 Did all patients receive the same reference standard??	Yes	
4.4 Were all patients included in the analysis?	Yes	
RISK OF BIAS:<br>Could the patient flow have introduced bias? (High, Low or Unclear)	LOW	
